# Preconception factors associated with postnatal mental health and suicidality among first-time fathers: results from an Australian Longitudinal Study of Men’s Health

**DOI:** 10.1007/s00127-023-02421-3

**Published:** 2023-01-28

**Authors:** Rebecca Giallo, Karen Wynter, Grace McMahon, Monique Seymour, Alison Fogarty, Amanda Cooklin, Liana Leach, Lauren M. Francis, Elisabeth Duursma, Jacqui A. Macdonald

**Affiliations:** 1 Centre for Social and Early Emotional Development, School of Psychology, Faculty of Health, Deakin University, Geelong, Australia; 2 Murdoch Children’s Research Institute, Parkville, Australia; 3 Centre for Quality and Patient Safety, School of Nursing and Midwifery–Western Health Partnership, Institute for Health Transformation, Deakin University, Geelong, Australia; 4 LaTrobe University, Bundoora, Australia; 5 National Centre for Epidemiology and Population Health, Australian National University, Canberra, Australia; 6 University of Wollongong, Wollongong, Australia; 7 Centre for Adolescent Health, Murdoch Children’s Research Institute, Parkville, Australia; 8 Department of Paediatrics, University of Melbourne, Parkville, Australia

**Keywords:** Fathers, Postnatal mental health, Preconception mental health, Suicidal ideation

## Abstract

**Purpose:**

Prospective evidence about men at risk of postnatal difficulties is rare–particularly for postpartum suicidal ideation. This study aimed to determine the extent to which first-time fathers reported depressive symptoms and suicidal ideation and behaviours in the first postnatal year, and to identify preconception risk factors for postnatal mental health difficulties.

**Methods:**

Secondary analysis of data from The Ten to Men Study–Australia’s population-based prospective study of men’s health was conducted. Participants were 205 men who became first-time fathers in the 12 months prior to wave 2 (2015/16). Regression analyses were used to ascertain preconception (mental and physical health, lifestyle) and demographic factors associated with postnatal depressive symptoms.

**Results:**

Postnatally, 8.3% of fathers reported moderate to severe depressive symptoms, 5% had suicidal thoughts, 3% had plans, and less than 1% had attempted suicide. Preconception depressive symptoms was the only factor significantly associated with postnatal depressive symptoms.

**Conclusion:**

The transition into fatherhood is marked with significant psychological distress for some men. These results suggest that mental health screening and support in the preconception period is crucial to supporting the mental health of new fathers.

## Introduction

The mental health care needs of men continue to be pervasively neglected in perinatal and community child health care settings, despite accumulating evidence that approximately 10% of men experience postnatal mental health difficulties [1–3] and 6% report suicidal ideation [4, 5]. Fathers’ mental health difficulties have been associated with poor maternal mental health [2], suboptimal parenting behaviours [6], and adverse developmental outcomes for children [7]. Early detection of men at risk of postnatal mental health difficulties may lead to improved opportunities for mental health care and support at a life stage when men might be more receptive to help [8–10].

Research into factors associated with men’s postnatal mental health difficulties has primarily been cross-sectional, identifying couple relationship difficulties, poor partner mental health, and limited social support as key risks [11–13]. Although a past history of mental health problems has also been identified as a key risk factor, this evidence is primarily based on men’s retrospective reports [13]. In the few longitudinal studies with data prior to men’s transition to fatherhood, men with a history of mental health difficulties in adolescence and young adulthood were found to be over four times more likely to experience antenatal mental health difficulties [14], and over five times more likely to report postnatal depressive symptoms [15]. We know of no prospective research identifying preconception risk factors for suicidal ideation in new fathers.

Although these studies reinforce the importance of considering preconception mental health difficulties for men, few studies have examined the extent to which a broader range of preconception psychosocial factors (i.e., lifestyle behaviours, stressful life events) are associated with postnatal mental health difficulties. From a life course social determinants of health perspective, this evidence is needed to inform both the development of tailored approaches to preconception care before men become fathers, and perinatal health care for men as they transition into fatherhood.

Using prospective data from Australia’s national longitudinal study of men’s health, we aimed to determine the extent to which first-time fathers reported depressive symptoms and suicidal ideation and behaviours in the first postnatal year. We also sought to identify preconception risk factors associated with postnatal depressive symptoms and suicidal ideation. We focused on risk factors that general practitioners/physicians (GP) and other health professionals are well placed to identify among men when providing preconception or prenatal care. These include psychosocial (i.e., history of mental health difficulties, stressful life events, partner violence), physical health (i.e., health problems), and lifestyle (i.e., alcohol and substance misuse) factors.

## Methods

### Study design and participants

Data were drawn from the Ten to Men Study, the Australian Longitudinal Study on Male Health [16–18]. Ethics approval was obtained from the University of Melbourne Human Research Ethics Committee and the Australian Government Department of Health. The target population was boys and men (aged 10–55 years) who were Australian citizens or permanent residents, living in private households in urban and regional areas across Australia. The study design and sample information are detailed elsewhere [16, 17]. Briefly, a stratified multi-stage cluster random sample design was used. Separate cluster samples were drawn from geographical strata representing major cities, and inner and outer regional areas, with oversampling of regional areas to increase the representation of males living in these areas. Over 45,510 males living in the sampled households were invited to participate between 2013 and 2014. A total of 15,988 boys and men participated at wave 1 (baseline), and they were followed up at wave 2 (2015–2016). The response rate at wave 2 was 75%. The current study used data from men in the adult cohort who indicated that they had become a father within the 12 months prior to wave 2 data collection (*n = *205).

Ten to Men fieldworkers made three in-person visits to each sampled household to recruit eligible males. Interested males were provided with study information, a consent form, hardcopy questionnaires, and privacy envelopes. Written informed consent was obtained from all participants and paper questionnaires were completed. The questionnaires covered broad topics including socio-demographic characteristics, mental health and wellbeing, physical health, health behaviours, socio-contextual information, and knowledge and use of health services.

### Measures

Demographic information at study enrolment included age, Aboriginal and/or Torres Strait Islander origin, country of birth, main language spoken at home, highest level of education, in paid employment, and relationship status (partnered or not). Additionally, the Modified Monash Model geographical classification was used to define whether the area in which a participant lived was city, rural, remote or very remote based on the size of the local town or city [19]. This was dichotomised into metropolitan areas and rural, remote or very remote areas in analyses. Age, English spoken at home, and education were included in the analyses as indicators of economic status. Metropolitan and rural/remote status was also included given disparities in access to health care in rural areas and some evidence of higher mental health difficulties and suicidality among men living in rural areas [20].

*Depressive symptoms* were assessed using the Patient Health Questionnaire-9 (PHQ-9; [21] at both waves 1 and 2. Nine items asked about the extent to which participants were bothered by depressive symptoms (e.g., little pleasure in doing things, feeling depressed/hopeless) in the last 2 weeks on a scale ranging from 0 = ‘not at all’ to 3 = ‘nearly every day’. The scores are summed, with scores between 0 and 4 indicating no or minimal depression, 5–9 mild depression, 10–14 moderate depression, 15–19 moderately severe depression, and 20–27 severe depression. The PHQ-9 has well established validity [22], and good internal consistency in the analytic sample (Cronbach’s α = 0.83 at wave 1 and 0.89 at wave 2).

*Preconception estimates of suicidal ideation and behaviours* was assessed at wave 1 using two self-report items from the Youth Risk Behaviour Survey [23]; ‘Have you ever tried killing yourself?’ and ‘Have you ever made a plan about how you would kill yourself?’, and a single item from the Longitudinal Study of Australian Children [24]; ‘Have you ever seriously thought about killing yourself?’. To obtain postnatal estimates of suicidal ideation and behaviours at wave 2, all three questions were asked again but in regards to the past 12 months. Participants were asked to respond ‘yes’ or ‘no’ to each item.

*Stressful life events* were assessed at both waves using 24 items selected from either the Australian Longitudinal Study on Women’s Health (ALSWH; [25] or the Social Readjustment Rating Scale (SRRS; [26]. Participants were asked to indicate ‘yes’ or ‘no’ to if they had experienced any of the listed stressful life events in the past 12 months. Events included personal injury or illness, divorce or marital separation, conflict between family, death of spouse, moving house, difficulty finding a job, natural disaster or house fire, legal troubles or court case. Items were scored by summing all the ‘yes’ responses, where higher scores indicate the presence of more stressful life events.

*Financial difficulties* were assessed using items from the Australian Bureau of Statistics [27]. Participants were asked if any of the following had happened over the past 12 months because they were short of money: (a) could not fill or collect prescription medicine, (b) could not get a medical test, treatment or follow-up that was recommended by a doctor, (c) limited how much fruit and vegetables you ate, (d) could not pay electricity, gas or telephone bills on time, (e) could not pay the mortgage or rent on time, and (f) asked for financial help from friends and family. Respondents were asked to indicate ‘yes’ or ‘no’ to each item, and endorsement of one or more was identified as experiencing financial difficulties (0 = no financial difficulties; 1 = one or more financial difficulties).

*Engagement in violence or experience of violence* with a past or present partner was assessed using two items from the Comparing Heterosexual and Same Sex Abuse in Relationships survey instrument [28]. Participants were asked if they had ever (a) made a past or present partner feel frightened or anxious, (b) forced a partner to have sex, and (c) hit, slapped, kicked or otherwise physically hurt a partner when angry. They were asked to indicate ‘yes’ or ‘no’ to each item, and endorsement of one or more was identified as having used partner violence (0 = not used violence; 1 = used violence). Participants were also asked if a past or present partner had ever engaged in these three behaviours toward them. Similarly, they were asked to indicate ‘yes’ or ‘no’ to each item, and endorsement of one or more was identified as having experienced partner violence (0 = not experienced partner violence; 1 = experienced partner violence).

*Social support *at waves 1 and 2 was assessed using the Emotional/Informational support subscale of the Medical Outcomes Study Social Support Survey (MOS; [29]. Eight items asked about the extent to which different kinds of support (e.g., “someone you can count on to listen to you when you need to talk”) are available if needed on a scale ranging from 1 = ‘None of the time’ to 5 = ‘All of the time’. The items are summed with higher scores indicating higher availability of social support. The MOS has excellent validity [29], and excellent internal consistency in the analytic sample (Cronbach’s α = 0.97).

*Lifestyle behaviours, physical health conditions and health service use* were assessed at waves 1 and 2 using self-reports. Life style behaviours were captured by asking participants if they currently smoke tobacco cigarettes, and have used cannabis or other illicit substances (i.e., ecstasy, cocaine or opiates) for non-medical purposes at least once in the past 12 months (‘yes’ or ‘no’). Harmful alcohol use was assessed using the Alcohol Use Disorders Identification Test (AUDIT; [30]. Participants were also asked if they had symptoms or been treated for a range of health conditions (e.g., asthma, diabetes, high cholesterol, high blood pressure) in the past 12 months (‘yes’ or ‘no’). Endorsement of one or more was identified as having a physical health condition (0 = no physical health condition; 1 = physical health condition). Health service use was assessed by a single item from the National Health Survey [31], where men were asked if they had consulted with a family doctor/General Practitioner (GP) for their own health in the past 12 months (‘yes’ or ‘no’).

### Data analysis

All analyses were performed using SPSS Version 22. Descriptive statistics for the sample demographics, preconception variables, depressive symptoms and suicidal thoughts, plan and attempts were generated (Aim 1). Bivariate and multivariate standard linear regression analyses were performed to identify preconception risk factors of men’s postnatal depressive symptoms (continuous scores on the PHQ-9; Aim 2). To select variables for inclusion in the multivariate models, we examined each potential predictor variable in a series of bivariate regressions. Predictor variables with significant bivariate associations with postnatal depressive symptoms at *p* < 0.05 and the demographic characteristics (father age, language spoken, high school education, metropolitan vs. rural or remote) were entered into the multivariate model. We were unable to conduct regression analyses to assess risk factors for suicidal ideation in the postnatal period due to the small number of fathers reporting suicidal ideation (*n = *10).

Missing data across all variables were minimal (5.2%) and missing completely at random as evidenced by Little’s MCAR test, *χ*^2^ (16) = 20.18, *p* = 0.212. Missing data for all descriptive and regression analyses were handled using multiple imputation. Fifty complete datasets were imputed using a multivariate normal model using all variables used in the analyses. Pooled estimates for all proportions and model parameter estimates were obtained using Rubin’s rule [32]. Multiple imputation in SPSS provides the following regression model estimates for pooled data: unstandardised B, *t* and p-values. For the remaining estimates (R^2^, F-statistic), the ranges across the multiply imputed datasets are reported.

## Results

### Sample characteristics

Of the 13,896 men in the adult cohort enrolled in the study, 9040 (65.1%) were already fathers and 6948 (50.0%) were not yet fathers at wave 1. By wave 2, 205 men indicated they had become fathers in the previous 12 months (analysis sample). Table 1 presents the sample characteristics for the analysis sample and shows that the majority of men were born in Australia, English speaking, had completed high school, were in paid employment and were partnered at wave 1.

**Table 1:** Characteristics of men who became fathers in the last 12 months at wave 2 (*N = *205)

	At study enrolment (wave 1) *n* (%)
Age in years, M (SD)	30.5 (5.6) (range: 18–49)
Country of birth–Australia	161 (78.9)
Aboriginal and/or Torres Strait Islander	8 (3.9)
English spoken at home	184 (89.8)
Completed high school	141 (68.8)
Paid employment	179 (89.1)
Relationship status – partnered	161 (79.3)
Living in metropolitan area	132 (64.4)

### Estimates of depressive symptoms and suicidal ideation and behaviours

With respect to depression, approximately 7.8% of men reported at least moderate symptoms in the preconception period (wave 1) compared to around 8.3% in the postnatal period (wave 2; see Table 2). Approximately 14% reported a lifetime history of suicidal ideation, plans and/or an attempt in the preconception period (wave 1), and 5% reported ideation, plans and/or an attempt in 12 months previous to the postnatal assessment (wave 2). In both the preconception and postnatal period, over 80% of men had consulted with their GP in the past 12 months. Approximately 71% of men with moderate to severe depressive symptoms and 80% of men who reported suicidal ideation in the postnatal period had seen a GP in the past 12 months.

**Table 2:** Preconception and postnatal depressive symptoms, suicidal ideation and behaviours, and psychosocial health, health behaviours and health service use at waves 1 and 2 (*N = *205)

	Preconception period (wave 1) *n* (%)	Postnatal period (wave 2) *n* (%)
Depressive symptoms, M (SD)^a^	3.8 (3.4–3.5)^a^	4.0 (3.8–4.0)^a^
Depression severity		
No or minimal (0–4)	137 (66.8)	134 (65.4)
Mild (5–9)	52 (25.4)	54 (26.3)
Moderate (10–14)	13 (6.3)	12 (5.9)
Moderately severe (15–19)	3 (1.5)	5 (2.4)
Severe (20–27)	–	1 (< 0.01)
Suicidal ideation and behaviour		
Thoughts	29 (14.1)^b^	10 (4.9)^c^
Plan	16 (9.0)^b^	6 (2.9)^c^
Attempt	6 (2.9)^b^	1 (0.5)^c^
Any	29 (14.1)^b^	39 (4.9)^c^
Psychosocial health, health behaviours and health service use		
Stressful life events, *M (SD)*^a^	2.2 (1.7–1.8)^a^	1.9 (1.7)
Financial difficulties	55 (26.8)	57 (27.8)
Partner violence–Experienced	56 (27.3)	–^d^
Partner violence–Used	47 (22.9)	–^d^
Social support, *M (SD)*^a^	78.6 (21.4–22.8)^a^	74.1 (23.9–24.2)^a^
Harmful/hazardous alcohol use	86 (42.0)	74 (36.1)
Current cigarette smoking	101 (49.3)	90 (43.9)
Marijuana use	40 (19.5)	35 (17.1)
Illicit substance use	25 (12.2)	27 (13.2)
Physical health condition/s	51 (24.9)	38 (18.5)
Consulted GP in last 12 months	167 (81.5)	168 (82.0)

^a^Range across multiply imputed datasets

^b^Lifetime

^c^Past 12 months

^d^Not available

### Predictors of men’s postnatal depressive symptoms

Table 3 presents the bivariate and multivariate linear regression analyses to identify predictors of men’s postnatal depressive symptoms using continuous PHQ-9 scores. At a bivariate level, the preconception variables (wave 1) associated with postnatal depressive symptoms were high depressive symptoms, suicidal ideation (life time), financial difficulties, being a victim of partner violence, and use of partner violence. These were entered into the multivariate model along with the demographic characteristics. This model significantly accounted for 28–32% of the variance in postnatal depressive symptoms as indicated by the range for *R*^2^ across the multiply imputed datasets (*R*^2^ = 0.28–0.32). The range for the *F*-tests was *F*(9, 195) = 8.50, *p* < 0.001 to *F*(9, 195) = 10.34, *p* < 0.001. Preconception depressive symptoms was the only factor significantly associated with postnatal depressive symptoms in this model.

**Table 3:** Pooled estimates for multiple linear regression analyses examining factors associated with men’s postnatal depressive symptoms (continuous PHQ-9 scores; *N = *205)

	Bivariate analyses			Multivariate analyses		
	*B*^a^	*t*	*p*	*B*^a^	*t*	*p*
Preconception mental health						
Depressive symptoms (PHQ-9)	0.58	8.64	< 0.001	0.55	7.89	< 0.001
Suicidal thoughts (lifetime)	2.22	2.83	0.005	0.70	0.98	0.325
Preconception psychosocial, physical health, and lifestyle factors						
Stressful life events	0.21	1.28	0.200	–	–	–
Financial difficulties^b^	1.29	2.12	0.034	0.64	1.15	0.250
Partner violence–Experienced^b^	1.26	2.09	0.037	0.35	0.62	0.534
Partner violence–Used^b^	1.82	2.87	0.004	1.05	1.73	0.084
Social support	− 0.02	− 1.45	0.147	–	–	–
Harmful alcohol use^b^	0.45	0.73	0.465	–	–	–
Current smoking^b^	0.31	0.04	0.969	–	–	–
Marijuana use^b^	− 0.38	− 0.53	0.596	–	–	–
Illicit substance^b^	0.87	0.91	0.366	–	–	–
Physical health condition/s^b^	− 0.23	− 0.38	0.707	–	–	–
Demographic characteristics						
Respondent age	− 0.01	− 0.21	0.833	0.0	0.11	0.911
English spoken at home	0.24	0.26	0.798	0.33	0.40	0.690
Completed high school	− 0.38	− 0.65	0.514	0.29	0.55	0.585
Living in a metropolitan area	− 0.60	− 1.06	0.288	0.26	0.05	0.960

^a^Pooled unstandardised beta estimates. All predictors assessed at wave 1. PHQ-9 = Patient Health Questionnaire-9

^b^Dichotomous variables (1 = no, 2 = yes)

## Discussion

This study is one of few prospective studies using national cohort data to investigate *preconception* risk factors for men’s postpartum mental health, which is critical to identifying opportunities for intervention and support prior to becoming fathers. We found that one in four fathers reported mild depressive symptoms in the postnatal period, and 8.3% reported moderate to severe depressive symptoms. Our finding is consistent with a meta-analytic prevalence estimate of 8.4% (95% CI 7.2–9.6) for postnatal depression among men [1]. Of particular note, our study found that 5% of fathers reported thoughts of suicide, made a plan for suicide, and/or attempted suicide in the 12 months prior to wave 2. This is consistent with an estimate of 4.8% in one of the only other studies reporting on men’s suicidal ideation and behaviour in the postnatal period using a diagnostic interview [5]. Although we cannot infer that the psychological distress among men in our study is due to the transition to fatherhood, our findings highlight that this is a stage of life when some men may experience mental health difficulties and that this is an opportune time to identify and support these men. This is of public health concern given that men’s postnatal mental health difficulties can have adverse consequences for fathers themselves and their families [2, 6, 7, 33].

Another key finding from our study is the stability in estimates for moderate to severe depressive symptoms at the preconception (7.8%) and postnatal time points (8.3%). This is consistent with studies indicating that men’s mental health may not substantially worsen as they transition into fatherhood, and that some fathers may experience a continuation or recurrence of earlier difficulties possibly triggered by the stressors of the postnatal period [14, 34, 35]. In support of this, we found that preconception depressive symptoms were predictive of future depressive symptoms in the postnatal period. Other preconception risk factors for postnatal depressive symptoms were identified in our bivariate analyses. These included experiencing financial difficulties, and being in a violent partner relationship either as a victim or user of violence. Taken together, these findings reinforce the importance of mental health promotion and care for men before men transition into fatherhood.

### Health promotion and prevention in the preconception period

Over the last decade there have been calls for a greater focus on the provision of preconception health care for men [8, 9, 36–38]. For example, the Australian National Men’s Health Strategy acknowledges that preconception health promotion and reproductive health issues warrant more attention in primary care health settings [39]. Given that the vast majority of men (80%) in our study reported seeing a general practitioner (GP) in wave 1 prior to having their first baby, the preconception period might be an opportune time to engage men about their mental health, lifestyle behaviour and reproductive concerns to prevent the onset and/or reoccurrence of mental health difficulties. However, research indicates that GPs do not routinely engage men in discussions about preconception health [40, 41]. This is often due to health care providers’ lack of knowledge about men’s preconception health, sensitivity of the subject, and reproductive health being perceived as a female issue [40]. This suggests that opportunities to engage men about their mental health, suicidality and lifestyle behaviours, and offer preventative health care and support before they make the transition to fatherhood, are currently being missed.

### Early intervention and mental health care in the postnatal period

Early intervention to identify and support men experiencing mental health difficulties in the postnatal period is also needed. The estimates for postnatal depressive symptoms (8.3%) and suicidal ideation among men (5%) in our study are lower but still comparable to those for women, with meta-analytic estimates of 14% for maternal depressive symptoms [42] and 7% for suicidal ideation [43]. Yet, women remain the primary focus in maternity and maternal-child health services, and opportunities to identify fathers experiencing mental health difficulties are being missed [44]. Although midwives have indicated that they would like to engage fathers (and non-birthing partners) more actively in the provision of antenatal care, barriers include lack of knowledge about men’s health at this time and lack of time to engage men in appointments [45]. Another barrier is the perceived lack of referral pathways for fathers who are not ‘patients’ in antenatal care and are not covered by the same referral pathways as women accessing antenatal care services [46]. Given that the majority of men reporting moderate to severe depressive symptoms (71%) and suicidal ideation (80%) in the postnatal period had seen a GP in the last 12 months, identification and mental health care opportunities for fathers also exist in primary health care. The extent to which new fathers in primary health services are routinely asked about their mental health and provided with support remains unclear.

### Limitations

Before further considering the implications of our study, there are several limitations to note. First, the study response rate was low, with 35% of all eligible males contacted enrolling in the study [18]. It is possible that men experiencing significant mental health difficulties, and those who speak English as a second language or experience socio-economic disadvantage may have chosen not to participate in the study. Further to this, although study retention was excellent at 75% and we accounted for non-response at wave 2 using multiple imputation methods, attrition at wave 2 was associated with depressive symptoms, cigarette smoking, high alcohol use and illicit drug use [47]. Therefore, due to potential non-response and selective attrition bias, the estimates of depressive symptoms and suicidal ideation in our study are potentially conservative.

Second, whilst secondary analysis of existing population-based data is a strength of this study, the Ten to Men Study was not designed or powered specifically with new fathers in mind. Therefore, the analysis sample for our study was relatively small with 205 men indicating they had become a father in the 12 months prior to the wave 2 data collection. No information was collected about when their child was born, so it was not possible to determine how many months had passed since the birth of their child, and whether this was associated with mental health difficulties. Given the small sample size of fathers as well as low prevalence of suicidal ideation and behaviours, it was not possible to conduct analyses to identify risk factors for suicidal ideation in the postnatal period.

Third, a validated self-report measure of depressive symptoms was used. Given the potential for self-reporting bias, a diagnostic interview to assess depressive symptoms and other mental health difficulties would strengthen our understanding and confidence of the prevalence of mental health difficulties among men in early fatherhood. Further, there were no assessments of anxiety or stress symptoms in waves 1 and 2. This will be an important area for future research to investigate, as research suggests that anxiety and stress symptoms are more prevalent than depressive symptoms among men in the postnatal period [3, 4, 48]. Despite rich assessments of health and lifestyle behaviours in the preconception period, there were few assessments of men’s psychosocial context (relationship and family functioning) in the Ten to Men Study, limiting identification of risk factors related to family of origin and partner relationship history.

### Conclusion

Notwithstanding these limitations, this study underscores the importance of preconception and perinatal mental health care for men as they make the transition to fatherhood. Despite calls for comprehensive models of preconception and perinatal health care for men [8, 9, 38], they are not routinely offered this care in universal settings. Research and advocacy efforts are needed to: (a) increase awareness among GPs about the extent to which men experience depressive symptoms and suicidal ideation in early fatherhood, (b) generate the evidence-base for preconception and perinatal mental health interventions for new fathers, (c) increase confidence and capacity of the workforce to engage fathers, and identify and respond to their mental health needs, and (d) determine effective strategies to support the universal implementation of preconception and perinatal health care for men. Investing in prevention and early intervention efforts to optimise men’s mental health in early fatherhood is a critical step toward promoting the health and wellbeing of families and future generations.

## Data Availability

Data for this paper were drawn from the Australian Longitudinal Study on Male Health (*Ten to Men*). *Ten to Men* research data is the intellectual property of the Commonwealth of Australia. Research data are available from The Australian Government Australian Institute of Family Studies.

## References

[1] Cameron EE, Sedov ID, Tomfohr-Madsen LM (2016). Prevalence of paternal depression in pregnancy and the postpartum: an updated meta-analysis. J Affect Disord.

[2] Paulson J, Bazemore S (2010). Prenatal and postpartum depression in fathers and its association with maternal depression. J Am Med Assoc.

[3] Leach LS (2016). Prevalence and course of anxiety disorders (and symptom levels) in men across the perinatal period: a systematic review. J Affect Disord.

[4] Giallo R (2013). The psychological distress of fathers attending an Australian Early Parenting Centre for early parenting difficulties. Clincial Psychol.

[5] Quevedo L (2011). Risk of suicide and mixed episode in men in the postpartum period. J Affect Disord.

[6] Giallo R (2015). Trajectories of fathers’ psychological distress across the early parenting period: implications for parenting. J Fam Psychol.

[7] Sweeney S, MacBeth A (2016). The effects of paternal depression on child and adolescent outcomes: a systematic review. J Affect Disord.

[8] Garfield CF (2015). Supporting fatherhood before and after it happens. Pediatrics.

[9] Kotelchuck M, Lu M (2017). Father's role in preconception health. Matern Child Health J.

[10] Rominov H (2018). “Getting help for yourself is a way of helping your baby”: Fathers’ perceived support needs for mental health and parenting in the perinatal period. Psychol Men Masc.

[11] Giallo R (2013). Psychosocial risk factors associated with fathers' mental health in the postnatal period: Results from a population-based study. Soc Psychiatry Psychiatr Epidemiol.

[12] Giallo R, Cooklin A, Nicholson J (2014). Risk factors associated with trajectories of mothers' depressive symptoms across the early parenting period: an Australian population based longitudinal study. Arch Womens Ment Health.

[13] Wee KY (2011). Correlates of ante- and postnatal depression in fathers: a systematic review. J Affect Disord.

[14] Spry E (2018). Preconception prediction of expectant fathers' mental health: 20-year cohort study from adolescence. BJPsych Open.

[15] Thomson KC (2020). Adolescent antecedents of maternal and paternal perinatal depression: a 36-year prospective cohort. Psychol Med.

[16] Currier D (2016). The Australian Longitudinal Study on Male Health-methods. BMC Public Health.

[17] Spittal MJ (2016). The Australian Longitudinal Study on Male Health sampling design and survey weighting: implications for analysis and interpretation of clustered data. BMC Public Health.

[18] Pirkis J, Macdonald J, English DR (2016). Introducing Ten to Men, the Australian Longitudinal Study on Male Health. BMC Public Health.

[19] Australian Government Department of Health and Aged Care (2021) Modified Monash model. https://www.health.gov.au/health-topics/rural-health-workforce/classifications/mmm. Accessed 10 Aug 2022

[20] Fitzpatrick SJ (2021). Suicide in rural Australia: a retrospective study of mental health problems, health-seeking and service utilisation. PLoS ONE.

[21] Spitzer RL, Kroenke K, Williams JB (1999). Validation and utility of a self-report version of PRIME-MD: the PHQ primary care study. Primary care evaluation of mental disorders. Patient Health Questionnaire. JAMA.

[22] Kroenke K, Spitzer R, Williams J (2001). The PHQ-9: validity of a brief depression severity measure. J Gen Intern Med.

[23] Centers for Disease Control and Prevention (2011). Youth risk behaviour surveillance system: middle school survey.

[24] Australian Institute of Family Studies (2018). Growing Up in Australia: The Longitudinal Study of Australian Children (Wave 8 K Cohort Interview).

[25] Pachana NA, Brilleman SL, Dobson AJ (2011). Reporting of life events over time: methodological issues in a longitudinal sample of women. Psychol Assess.

[26] Holmes TH, Rahe RH (1967). The social readjustment rating scale. J Psychosom Res.

[27] Australian Bureau of Statistics (2003). Household expenditure survey and survey of income and housing costs.

[28] Donovan C (2005). Comparing heterosexual and same sex abuse in relationships (COHSAR Survey Instrument). Centre for gender and violence research, University of Bristol and Centre for Applied Social Science.

[29] Sherbourne C, Stewart A (1991). The MOS social support survey. Soc Sci Med.

[30] Bohn MJ, Babor TF, Kranzler HR (1995). The alcohol use disorders identification test (AUDIT): validation of a screening instrument for use in medical settings. J Stud Alcohol.

[31] Australian Bureau of Statistics (2007). National Health Survey 2007–08: module E-healthy lifestyles.

[32] Rubin DB (1987). Multiple imputation for nonresponse in surveys.

[33] Giallo R (2012). Mothers' and fathers' involvement in home activities with their childrenL psychosocial factors and the role of parental self-efficacy. Early Child Dev Care.

[34] Lowrie N (2022). Association of adolescent and young adult depression and anxiety with perinatal mental health in fathers: findings from an Australian Longitudinal Study. J Psychiatr Res.

[35] Leach LS (2014). New fatherhood and psychological distress: a longitudinal study of Australian men. Am J Epidemiol.

[36] Frey KA (2008). The clinical content of preconception care: preconception care for men. Am J Obstet Gynecol.

[37] Macdonald J (2021). Let's talk periconception health care with men. Med Today.

[38] O'Brien AP (2018). Men's preconception health: a primary health-care viewpoint. Am J Mens Health.

[39] Australian Government (2018). Commonweath Department of Health, National Men's Health Strategy 2020–2030.

[40] Hogg K (2019). Men's preconception health care in Australian general practice: GPs' knowledge, attitudes and behaviours. Aust J Prim Health.

[41] Frey KA, Engle R, Noble B (2012). Preconception healthcare: what do men know and believe?. J Men's Health.

[42] Liu X, Wang S, Wang G (2022). Prevalence and risk factors of postpartum depression in women: a systematic review and meta-analysis. J Clin Nurs.

[43] Xiao M (2022). Prevalence of suicidal ideation in pregnancy and the postpartum: a systematic review and meta-analysis. J Affect Disord.

[44] Macdonald JA (2021). How are you sleeping? Starting the conversation with fathers about their mental health in the early parenting years. J Affect Disord.

[45] Rominov H (2017). Midwives’ perceptions and experiences engaging fathers in the perinatal period. Women Birth.

[46] Wynter K (2021). Midwives' experiences of father participation in maternity care at a large metropolitan health service in Australia. Midwifery.

[47] Australian Institute of Family Studies (2017) Ten to Men: the Australian Longitudinal Study on Male Health Technical Report #6 January 2017, A.I.o.F. Studies, Editor, Australia.

[48] Wynter K, Rowe H, Fisher J (2013). Common mental disorders in women and men in the first six months after the birth of their first infant: a community study in Victoria, Australia. J Affect Disord.

